# Fluorescence imaging reversion using spatially variant deconvolution

**DOI:** 10.1038/s41598-019-54578-0

**Published:** 2019-12-02

**Authors:** Maria Anastasopoulou, Dimitris Gorpas, Maximilian Koch, Evangelos Liapis, Sarah Glasl, Uwe Klemm, Angelos Karlas, Tobias Lasser, Vasilis Ntziachristos

**Affiliations:** 1 Chair of Biological Imaging and TranslaTUM, Technical University Munich, Munich, 81675 Germany; 2 Institute of Biological and Medical Imaging, Helmholtz Zentrum München, Neuherberg, 85764 Germany; 3 Computer Aided Medical Procedures, Technical University Munich, Garching, 85748 Germany

**Keywords:** Biomedical engineering, Imaging and sensing

## Abstract

Fluorescence imaging opens new possibilities for intraoperative guidance and early cancer detection, in particular when using agents that target specific disease features. Nevertheless, photon scattering in tissue degrades image quality and leads to ambiguity in fluorescence image interpretation and challenges clinical translation. We introduce the concept of capturing the spatially-dependent impulse response of an image and investigate Spatially Adaptive Impulse Response Correction (SAIRC), a method that is proposed for improving the accuracy and sensitivity achieved. Unlike classical methods that presume a homogeneous spatial distribution of optical properties in tissue, SAIRC explicitly measures the optical heterogeneity in tissues. This information allows, for the first time, the application of spatially-dependent deconvolution to correct the fluorescence images captured in relation to their modification by photon scatter. Using experimental measurements from phantoms and animals, we investigate the improvement in resolution and quantification over non-corrected images. We discuss how the proposed method is essential for maximizing the performance of fluorescence molecular imaging in the clinic.

## Introduction

Fluorescence imaging has been widely used for assessing the distribution of fluorescent agents in tissues^1–5^ and it is increasingly considered in clinical applications^6–10^. The administration of agents with specificity for disease biomarkers opens up the field of Fluorescence Molecular Imaging (FMI), which has led to marked improvements in guiding surgery^11–16^ and detecting cancer early^17^ in pilot clinical studies.

Despite this early demonstration of clinical potential, the imaging performance of the method is limited by the interaction of photons with the tissue optical properties, i.e. scattering and absorption. This photon-tissue interaction degrades image quality, reduces the resolution in the optical image and negatively impacts the overall quantification accuracy and may lead to misinterpretation of fluorescence distribution in tissues^18–20^. In clinical studies, the dependence of the fluorescence image on optical properties may lead to false positives and false negatives^1,12^. Moreover, blurry fluorescence images lead to uncertainty about the precise delineation of tumor margins and may impair the decision-making process during surgery, increasing the risk of incomplete tumor resection or excessive removal of healthy tissue.

While these FMI limitations are generally known^1,12,20^, there has been limited methodology proposed to improve on the dependence of fluorescence images on optical properties and/or light diffusion^21,22^. Computational methods have been considered for improving decision making during surgery by estimating tumor borders based on adaptive thresholds^23^, but they operate on uncorrected fluorescence images that are subject to image degradation due to photon diffusion. Another promising strategy performs FMI in the so-called second near-infrared optical window (NIR-II, 1000–1400 nm)^24,25^, due to the known reduction of photon scattering with increasing wavelength. While signals collected in NIR-II experience less scattering than in the NIR, the image dependence on tissue optical properties does not fundamentally change in the NIR-II. Scattering remains a source of resolution reduction and tissue absorption is generally greater in the NIR-II than in the NIR-I, which makes images more strongly dependent on absorption depending on tissue type and experimental conditions. Structured illumination methods have been suggested for improving the contrast and resolution of fluorescence imaging by selectively exciting specific spatial modes in tissue that favor the target imaged^26,27^. Demonstrated in phantoms and animals, this class of methods generally requires scanning different spatial patterns in tissue. To address this problem, a method that retrieves optical properties as a single snap-shot has been suggested^21^. Fluorescence images are then corrected using a computational model, which however does not take into account spatial dependencies across the image, but only corrects the intensity of the images on a pixel-to-pixel basis.

Deconvolution is more commonly used for image enhancement in a variety of applications spanning astronomy^28^, computer vision^29^, widefield fluorescence microscopy^30^ and confocal microscopy^31^. The deconvolution procedure treats the acquired image as the convolution of the original image (ground truth) with a degradation kernel. Then, the convolution process is computationally “inverted”, leading to the original image. Deconvolution operations typically work on the assumption that image modification is due to imperfections of the measurement device employed. Typically, in optical systems, a space- and time-invariant kernel is employed, termed a point spread function (PSF), which is dependent on the hardware properties of the device utilized.

Even though deconvolution is well established in other imaging fields, its application to clinical fluorescence images has not been widely considered^32^, because FMI degradation depends primarily on the optical properties of the tissue imaged and not on the acquisition device. For example, the resolution of a charge-coupled device (CCD) camera can be on the order of a few tens of microns, such that the physical limits of photon diffusion result in an image with a resolution at least one order of magnitude worse. Typically, tissues in the surgical field of view vary in the amount of scattering and absorption and the images collected may be further affected by bleeding and other fluids. Therefore, the FMI degradation kernel is spatially dependent on the tissue imaged and generally not known, limiting the application of deconvolution to FMI images. In light of the expanding clinical applications of FMI, there remains a critical unmet need to develop approaches that revert the effect of tissue on FMI performance^20^ and lead to accurate fluorescence images, overcoming the aforementioned challenges.

We hypothesized that the combination of two novel concepts relating to capturing and reverting the effect of optical properties would lead to improving FMI performance. The first concept relates to spatially resolving the effects of tissue on the fluorescence image. The second concept relates to using this spatially dependent information to correct fluorescence images, enabling for the first time the application of deconvolution to FMI. Uniting these two concepts, we introduce herein Spatially Adaptive Impulse Response Correction (SAIRC), a novel framework that uses spatially variable kernels in an adaptive fashion to correct the fluorescence images. The inventive step of the method is the use of an inexpensive Digital Light Processing (DLP) projector that excites spatial impulse responses (PSFs) across the field of view imaged by projecting illumination points (delta functions) onto tissue. SAIRC captures these spatial impulse responses of tissue and assembles them in a matrix of spatially varying kernels that drives a variant deconvolution scheme. We developed spatially adaptive deconvolution based on a modified fast Lucy-Richardson algorithm^33^. In this paper, we describe studies to demonstrate the SAIRC principle in phantoms and animal measurements, and we discuss how SAIRC can be implemented in clinical FMI systems for real-time applications.

## Results

**Figure 1:**
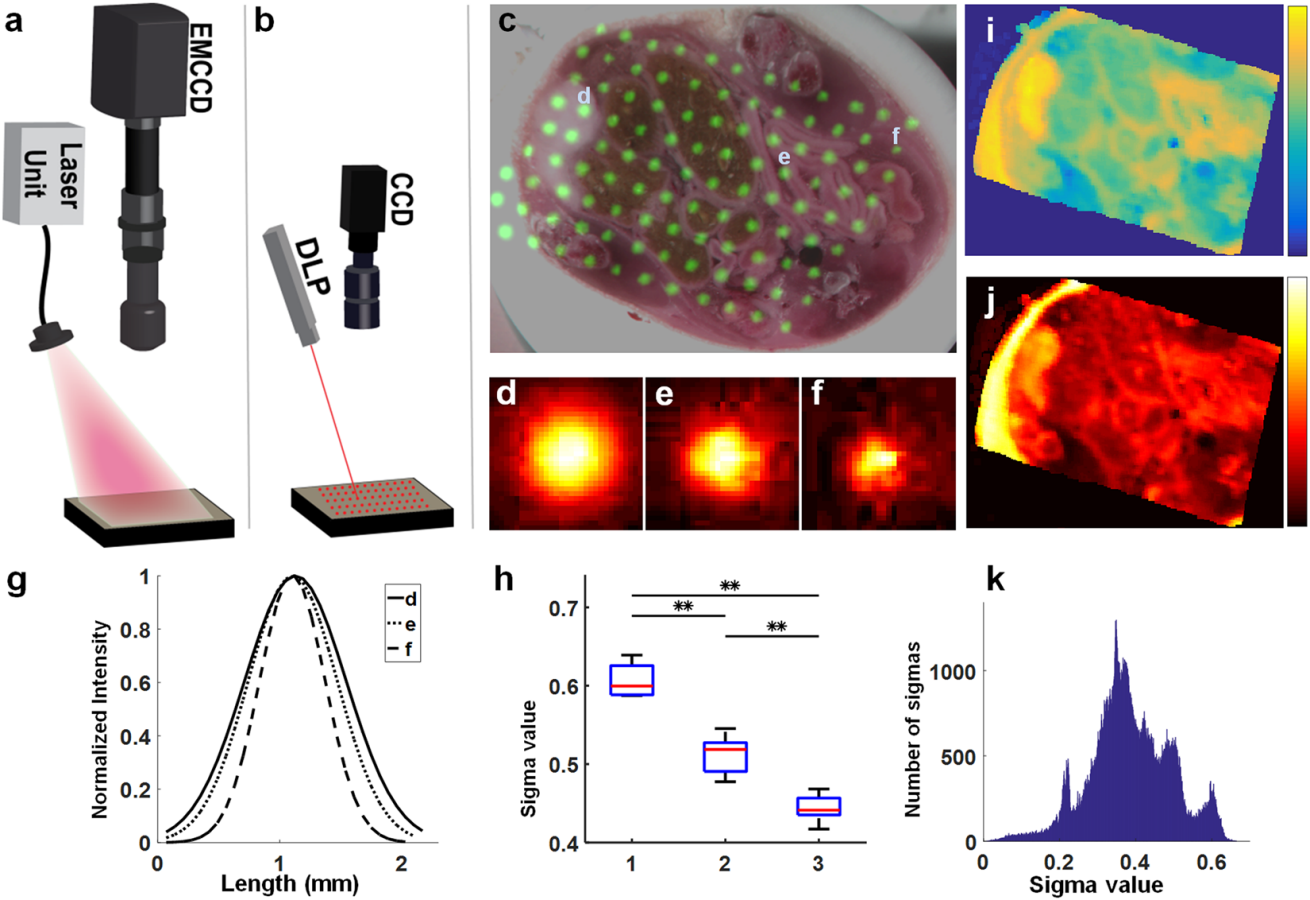
Schematic of the SAIRC process to enhance fluorescence imaging. Schematic system configuration for (**a**) fluorescence image acquisition and (**b**) scanning with the projector. (**c**) Color image of tissue from a mouse with an HCT116 tumor embedded in OCT (Optimal Cutting Temperature) compound after several cuts, overlaid (green pseudocolor) with some of the acquired impulse response images from the scanned area. The small panels underneath show images of spots from different areas: (**d**) tumor; (**e**) intestine; (**f**) muscle. Spots d, e and f are indicated in panel (c). (**g**) Comparison of the normalized profile of Gaussian curves fitted to the points d, e, and f. (**h**) Distribution of the sigma values fitted to five random spots chosen from areas 1, 2, 3 around points d, e, f. (**i**) Two-dimensional sigma map, extracted from the fitted Gaussians for the whole scanning area. (**j**) Sum of all the acquired impulse response images. (**k**) Histogram of sigma values binned into *n* bins, each of which contained the same number of sigma values. **p < 0.01.

Phantoms and tissues were imaged using a previously described FMI system (Fig. 1a) used in clinical studies in open surgery^11,23,34^ or endoscopy^17,35–39^ and illuminated by a spatial impulse response, using the red channel of a DLP projector (wavelength, 624 ± 18 nm) (Fig. 1b), that was scanned throughout the tissue. The broadening of the impulse responses emitted at tissues was captured by a monochromatic camera (Fig. 1c). Different parts of tissue from an HCT116 tumor-bearing mouse demonstrated different broadening, which is characteristic of different optical properties. As an example, highly scattering cancerous tissue (spot d; Fig. 1c) shows higher broadening (Fig. 1d) compared to an impulse response collected from intestinal and muscle tissue (spots e, f; Fig. 1c) that show less broadening (Fig. 1e,f). At each of the three spots studied herein, we fitted a 2D symmetrical Gaussian function (Fig. 1g) to better illustrate the differences in broadening and optical properties among the different tissue types.

We extracted the standard deviation (sigma) from each fitted Gaussian for random spots (spots d-f; Fig. 1c and for four spots in each of the areas 1 (cancer), 2 (intestinal), and 3 (muscle), where each of the areas was defined as having relatively uniform optical properties. The sigma value distribution (Fig. 1h) showed that sigma varied only slightly within each area, and that the sigma distribution varied significantly across different areas according to Mann-Whitney test (Fig. 1h). After scanning the entire tissue surface with the projected DLP point and extracting the sigma value from each Gaussian fitting (see Methods; Retrieval of the kernels), we assembled a 2D sigma map (Fig. 1i) that displays the spatial variation in tissue diffusion. The sum of all the projected points (Fig. 1j) resembles the illumination of the tissue with a homogeneous planar source.

The sigma map (Fig. 1i) was also employed to assemble the PSFs that were used for variant deconvolution of the blurred fluorescence image of the tissue. From the histogram of the sigma map (Fig. 1k), the sigma values were binned into *n* equally sized bins, and the lowest value of each bin was used to construct a Gaussian kernel. This kernel was deconvolved with the raw blurry fluorescence image using a modified Lucy-Richardson deconvolution scheme. The deconvolution process was repeated for every binned kernel, and the final corrected fluorescence image was constructed using a weighted summation of all the deconvolved images. This binning process minimized the deconvolution time with multiple kernels.

**Figure 2:**
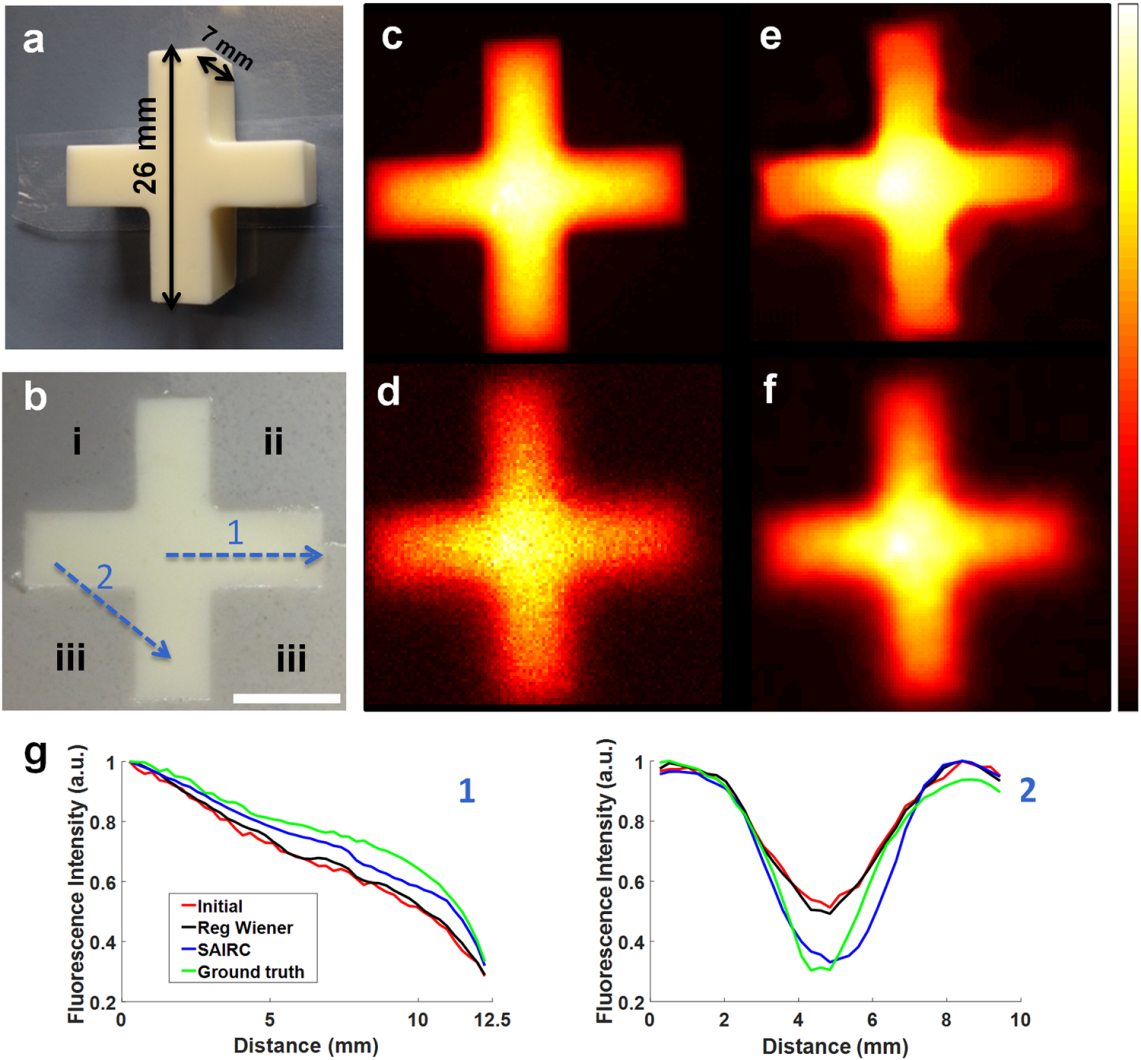
SAIRC improvement in fluorescence image quality of a phantom. (**a**) Color photograph of the phantom surrounded by air. (**b**) Color photograph of the phantom surrounded by three areas (i, ii, iii) with different optical properties based on different ink concentrations (see main text). Scalebar: 10 mm. (**c**) Ground truth fluorescence image. (**d**) Raw fluorescence image of panel (b). (**e**) Fluorescence image after the implementation of SAIRC. (**f**) Fluorescence image after the implementation of regularized Wiener deconvolution. (**g**) Fluorescence intensity profiles 1 and 2 from panel (b). Intensities 1 and 2 are normalized to 0–1. Initial: Raw Fluorescence Image, Reg Wiener: regularized Wiener deconvolution, SAIRC: Deconvolved Image with spatially adaptive kernels.

We designed a phantom to explore basic parameters of the proposed SAIRC framework and to determine SAIRC performance in acquired images containing substantial noise (Fig. 2). A cross-shaped agar phantom containing Alexa Fluor 750 and dairy cream (μ_s_ = 150 cm^−1^, μ_a_ = 0.05 cm^−1^) (Fig. 2a) was embedded into a background agar medium consisting of areas with different optical properties (Fig. 2b). The background medium consisted of area i (μ_s_ = 150 cm^−1^, μ_a_ = 2.32 cm^−1^), area ii (μ_s_ = 150 cm^−1^, μ_a_ = 1.16 cm^−1^) and area iii (μ_s_ = 150 cm^−1^, μ_a_ = 0.58 cm^−1^), each formulated with different concentrations of black ink (see Methods; Targets; Phantom). Because of their different optical properties, these areas should modify the projected points differently.

Figure 2c was considered the ground truth image (see Methods; System and data acquisition; Ground truth images) and shows the fluorescence image of the phantom in Fig. 2a which was surrounded by air. Figure 2d shows the raw fluorescence image of the phantom of Fig. 2b. We can observe the difference between the edges of area i and the edges of areas ii and iii: the edges in area i appear better resolved as compared to areas ii and iii. Instead,diffusion dominates in areas ii and iii, where the phantom’s edges appear more blurry. Figure 2e shows the results of the SAIRC process. Then, the results of SAIRC were compared with those obtained using regularized Wiener deconvolution (see Methods; Data Processing; Evaluation of Performance), which applies regularization and a fixed PSF (Fig. 2f). As can be seen, the SAIRC-corrected image (Fig. 2e) was better resolved than the original fluorescence image (Fig. 2d) and the image obtained from the Wiener deconvolution method (Fig. 2f), while it resembled the ground truth image (Fig. 2c) more closely. The edges were adequately restored from round to rectangular, recovering the original shape of the phantom; in addition, the noise artifacts were suppressed by the use of the regularization term λ, and the shape was smoothed out. It was necessary to use a relatively high λ value, reflecting the greater amount of noise in the acquired image.

To quantify the ability of SAIRC to restore image sharpness and edge quality, we drew line fluorescence intensity profiles across two different regions of the phantom (dotted blue lines 1 and 2 in Fig. 2b). In Fig. 1g we compared the intensity profiles in the raw, SAIRC, regularized Wiener and ground truth images, and we observed that the SAIRC profile was closest to the ground truth image for both lines. Furthermore, we assessed the image sharpness by calculating the Brenner gradient (see Methods; Data Processing; Evaluation of Performance), which was 0.0018 for the initial image, 0.0022 for the SAIRC-corrected image and 0.0010 for the regularized Wiener image. Consequently, the SAIRC-corrected image was 22% sharper than the initial image. The Root Mean Square (RMS) difference from the ground truth image was estimated to be 0.0621 for the initial image, 0.0445 for the SAIRC-corrected image, and 0.0457 for the regularized Wiener corrected image. This indicates that SAIRC increased similarity to the ground truth image by 28% compared to the raw image.

Encouraged by these results in phantoms, we tested our SAIRC algorithm by imaging abdominal cryosections of mouse bearing 4T1 tumor injected with the tumor-targeting fluorescence agent Integrisense 750, or mice bearing HCT116 tumors expressing the near-infrared fluorescent protein iRFP720.

**Figure 3:**
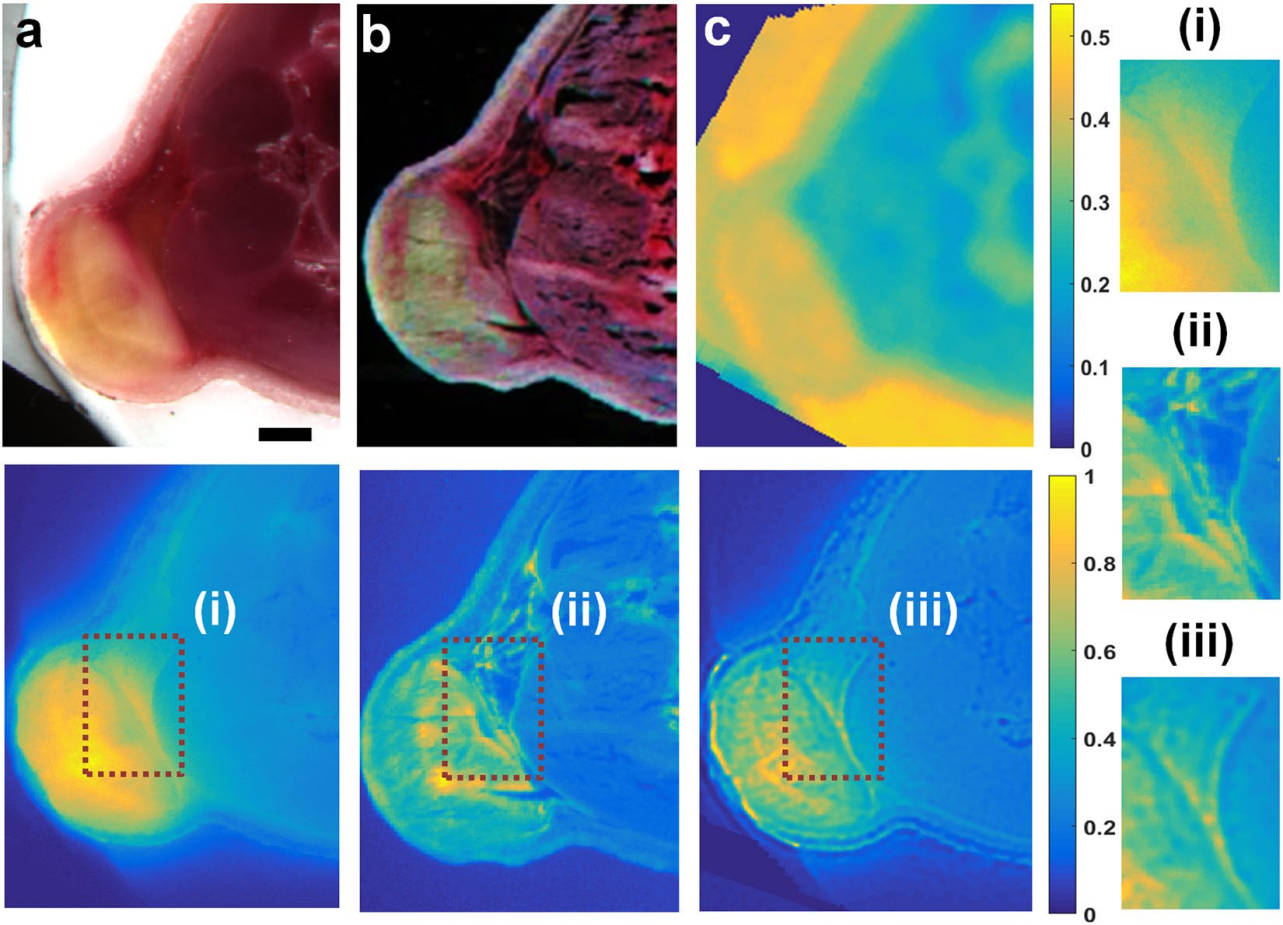
SAIRC improvement in the quality of fluorescence images of a mouse containing a 4T1 tumor injected with Integrisense 750. (**a**) Upper: color image of the back of a mouse bearing a 4T1 tumor after embedding in OCT compound. Lower: the corresponding raw fluorescence image of the tissue block. Scalebar: 1 mm. (**b**) Upper: color image of a thin slice (50 μm) from the OCT-embedded tissue block in front of a black background. Lower: the corresponding fluorescence image of the thin slice, which served as the ground truth image. (**c**) Upper: sigma map acquired from the scanning process (color bar in mm). Lower: corrected fluorescence image after the implementation of SAIRC. (i–iii): Zoomed-in areas of the regions of interest in the lower images in panels (a–c).

Figure 3 shows the performance of the SAIRC method for imaging a mouse bearing a 4T1 tumor. Upper panels of Fig. 3a,b depict color images of the OCT-embedded tissue block and a thin slice from the block, respectively. Lower panels of Fig. 3a,b show the corresponding raw fluorescence images. The fluorescence image of the thin slice (Fig. 3b) was considered the ground truth, due to its diffusion-free thickness. Comparison of the raw fluorescence image in Fig. 3b with the SAIRC-corrected fluorescence image (lower panel in Fig. 3c) shows that deconvolution process by using the acquired sigma map (upper panel of Fig. 3c) mitigated the effect of light diffusion on image quality, providing higher resolution, sharpness and resemblance to the ground truth image. Comparison of zoomed-in regions i, ii and iii further revealed how SAIRC correction clarified details in the fluorescence image, bringing it closer to the ground truth image. In fact, SAIRC was associated with a doubling of sharpness, as quantified with the Brenner gradient (0.0003 for Fig. 3a vs 0.0006 for Fig. 3c). SAIRC also increased similarity to the ground truth image by 37%, as quantified with RMS (Root Mean Square) (0.1918 for Fig. 3a vs 0.1402 for Fig. 3c). Some discrepancies between the corrected image and the thin slice can be attributed to tissue distortion during the cutting process.

**Figure 4:**
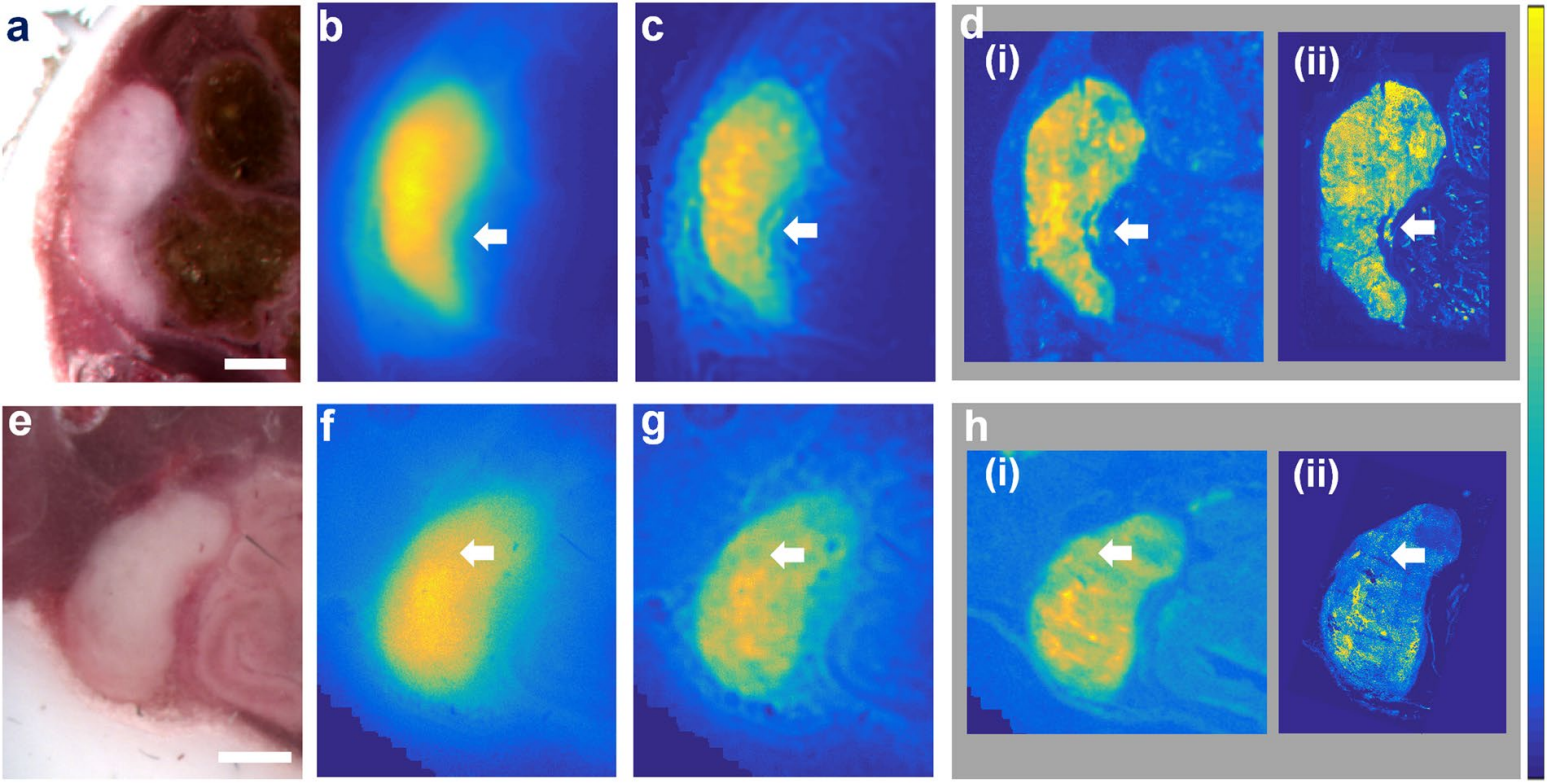
SAIRC improvement in quality of fluorescence images of mice bearing HCT116 tumors expressing IRFP720. The upper row corresponds to one mouse, while the lower row corresponds to a second mouse. (**a**,**e**) Color images of the abdominal area, after embedding in OCT compound. Scalebar: 1 mm. (**b**,**f**) Raw fluorescence image of the tissue block in panels (**a**) and (**e**). (**c**,**g**) Corrected image after the implementation of SAIRC (**d**,**h**). (**i**) Fluorescence image of a thin slice (50 μm) of the tissue block obtained under the same conditions as the block image. (**ii**) Stitched images of the thin slices obtained from a commercial fluorescence microscope. White arrows indicate tumor features that become clearly distinguishable only after SAIRC correction.

We observed similar improvements in image quality when we repeated the imaging experiments using tissue from two mice bearing HCT116 tumors expressing iRFP720 (Fig. 4). In this case SAIRC correction (Fig. 4c,g) brought the fluorescence images closer to the ground truth image [Fig. 4d(i),h(i)] than the initial images (Fig. 4b,f). As an additional verification of our results obtained with our custom FMI set-up, we obtained stitched fluorescence microscope images of the thin slices. The resulting images [Fig. 4d(ii),h(ii)] were consistent with those from our in-house system. Some tumor characteristics, which are marked with white arrows, are not visible in the raw fluorescence image but are revealed after SAIRC correction.

## Discussion

In this work, we demonstrate SAIRC, a novel fluorescence image correction scheme, which accurately captures optical property variations across the entire imaging area and enhances the acquired image via an iterative deconvolution process. A point-like beam from a DLP scans the imaging area, and spatially variant deconvolution kernels are retrieved from every corresponding beam region in the image and fitted to symmetrical Gaussian curves. Then, a Lucy-Richardson deconvolution method is applied, in which a regularization parameter^33^ is used to suppress noise, and kernel spatial variability is binned in order to minimize computational time. The final corrected image is the result of a weighted sum of the deconvolution results with the binned kernels. We validate and demonstrate the efficiency of our method using custom-made phantoms and *ex vivo* tissues from tumor-bearing mice. SAIRC improved fluorescence image resolution and contrast, increasing resemblance to the ground truth image by up to 37% and doubling the sharpness. This approach may increase the sensitivity and accuracy of FMI in preclinical and clinical applications, including surgical guidance and endoscopy.

In this work, we captured the diffusion modification at each point on the tissue by scanning over the tissue and translating the impulse response from every location to a representative value. This allowed us to visualize how diffusion in the tissue modifies the projected kernel in a 2D map. This information captures the spatial heterogeneity of the tissue and imports this spatial variability into a correction method based on deconvolution.

SAIRC correction enhanced image quality and structural details in phantoms and tumor tissue in mice. We further showed that SAIRC can perform well even when the acquired image contains a large amount of noise, such as with a highly noisy fluorescence phantom image. In this case, a higher value for the regularization parameter was needed. Sharpness of phantom images was enhanced by up to 22% relative to the initial fluorescence image, based on Brenner gradient estimation, while there was a 28% improvement compared with the initial image. We reduced the regularization parameter when processing the images of mouse tissue, where noise was less. In this case, the sharpness of the tissue image increased two-fold, and accuracy improved by 37%. We obtained greater improvements in sharpness and accuracy with mouse tissue than with phantoms. This can be attributed to the lower quality of the initial raw phantom image, where noise was greater. We further demonstrated that SAIRC provides better enhancement than another deconvolution method that uses regularization and invariant PSF.

SAIRC has the potential to be used with different combinations of imaging systems, tissue types and near-infrared fluorescence agents. The current method can be used with a variety of fluorescence agents upon proper modification of the projector light source, with the only limitation being the light penetration depth from both the excitation light and the projected scanning point. However, this intrinsic limitation is at the same time a great advantage of SAIRC, as the acquired data are not further blurred due to light propagating deep into tissues. SAIRC has the potential to be applied in fluorescence surgical navigation, where it can provide better insight into tumor structure and surroundings (Figs. 3 and 4). This is very important, since more accurate imaging of tumor areas offers better tumor delineation during surgical navigation and better insight into tumor topology. We envision the translation of SAIRC into a clinical FMI environment, offering better tumor delineation and insights into tumor heterogeneity, especially since such application is mainly focused on surface-derived rather than depth-resolved information. To implement SAIRC into clinical FMI, which requires real-time visualization, the overall process of scanning reflected points, fitting, and deconvolution will need to be greatly accelerated. We are working towards improving the scanning speed by scanning with a grid of multiple points simultaneously and by using a parallel rather than serial regime to conduct fitting for the sigma extraction (see Supplementary Notes on “2. Faster scanning acquisition”).

The current method may be improved through more accurate kernel acquisition and better fitting. In this work we employed the raw resolution of the projector without any lens coupling in front, which limited the resolution of the acquired sigma map. By coupling the projector to a focusing lens system, it may be possible to increase the spot resolution, although this would reduce the field of view unless projectors with higher number of effective pixels are used. In addition, more sophisticated methods for fitting (e.g. summation of non-symmetrical Gaussians) and for weighing different deconvolution results may increase the accuracy of the final image, while scanning time can be reduced by projecting a grid rather than single points, as well as by using parallel programming. We anticipate that these planned SAIRC improvements will increase the achieved similarity to the ground truth image from 37% (rms value of the difference between the ground truth and corrected image) to a much higher value. We note that SAIRC should not be strongly affected by differences in light scattering by tissue at the two different wavelengths of excitation and emission (see Supplementary Notes on “1. SAIRC dependence on wavelength”). Nevertheless, we are currently working on implementing SAIRC with a laser-coupled projector that would enable a variety of wavelengths and thus minimize any possible influence on SAIRC due to the difference in tissue optical properties between excitation and detection wavelengths.

A dual-tracer method for imaging tumors *in vivo* has recently been described^40,41^, in which a targeted tracer and a non-targeted one with close but separable fluorescence emission peaks are used to estimate tracer uptake by cancerous tissue. It may be possible to combine this method with SAIRC to generate tracer distributions of even higher resolution and accuracy. This will require additional work on SAIRC: although the method can correct for diffusion effects on fluorescence image resolution and contrast, it does not correct for the effect of absorption on the intensity of the fluorescence signal. The captured kernel can take into account optical properties and so may allow correction of fluorescence intensity^42^, which we are currently exploring. In order to be more accurate for this correction we need to expand the SAIRC scheme with scanning acquisition with both excitation and emission wavelength. By scanning with the corresponding wavelengths for both tracers and integrating this information into the SAIRC framework, we will be able to make the fluorescence signal more accurate and linearly proportional to the concentration of targeted and non-targeted tracers, disengaging the spatial variations in the optical properties of both tracer images of the heterogeneous tissue. This is critical^43^ for the best performance of dual-tracer method. Then, the concentration and localization of the tracer can be estimated through a tracer compartmental model^44^.

In summary, we demonstrated here the ability of SAIRC to correct fluorescence image degradation resulting from light diffusion and thereby a step closer towards improvement of distribution uptake of endogenous or exogenous tracers used in several clinical and basic research imaging applications. Combining our method with the range of fluorescence agents available, some of which efficiently target specific tissues such as tumors, and a dual tracer scheme may lead to new possibilities for imaging pathophysiological processes and guiding surgery. By extending the limits of fluorescence imaging, our method may help promote its incorporation into high-end imaging set-ups for surgery, endoscopy and other preclinical and clinical applications.

## Methods

### Targets

#### Phantom

A negative mold of a cross with dimensions 26 × 26 × 7 mm was developed and a mixture of scattering, absorbing, fluorescent agent and base material was cast inside the mold. Agar (1%) was selected as the base material, dairy cream (30% fat) as scattering agent, and acrylic ink as absorbing material. Alexa Fluor 750 (Thermo Fisher Scientific, Waltham, USA) was used as the fluorescent agent. The final composition of the phantom was 4 nM of Alexa Fluor 750, 15% fat dairy cream and 1% agar (Fig. 1a).

To test the efficacy of our deconvolution method, three additional areas of different optical properties were created around the fluorescence area. The three areas were formed by successive filling, solidifying and removing of excess agar, ink and cream milk in a petri dish. All three areas contained 15% fat dairy cream. Area i also contained 0.25% ink; area ii, 0.125% ink; and area iii, 0.0625% ink (Fig. 2b).

#### Animal models

All animal procedures were performed in accordance with protocols approved by the Government of Upper Bavaria. The cryo-imaging experiments were performed with female NOD SCID Shorn mice (Envigo, Germany) approximately 8 weeks old. In two mice, 2 × 10^6^ HCT116 human colon cancer cells expressing iRFP720 (ATCC, CCL-247, transfected in-house) were injected orthotopically into the abdominal area. In a third mouse, 1 × 10^6^ 4T1 breast cancer cells (ATCC, CRL-2539) were injected orthotopically into the mammary fat pad. All three tumors were allowed to grow until they reached a length of approximately 8 mm. Then, the animal bearing the 4T1 tumor was injected intravenously with Integrisense 750 (2 nmol; PerkinElmer), and sacrificed 24 h later. Mice were frozen at −50 °C and cryosliced as described previously^45,46^ for imaging.

### System and data acquisition

#### Fluorescence imaging

For the acquisition of the fluorescence phantom image, an optical system that has been previously described^47^ was used. Briefly, a 750-nm, 300-mW Continuous Wave fiber-coupled diode laser (BWF2-750-0, B&W, Newark, USA) was employed for the excitation of the phantom and an NIR emission filter (ET810/90, Chroma, Rockingham, USA) for the emission. Fluorescence images were recorded using a fluorescence camera (512 × 512, DV897DCS-BV EMCCD, Andor, Belfast, Northern Ireland), whereas the reflectance images were captured using a 14-bit CCD grayscale camera (Grasshopper 3, PointGrey, Wilsonville, USA). The two images were automatically co-registered and the values of the sigmas were adjusted accordingly. Fluorescence images were automatically acquired using C++ software developed by our group^35^.

For mouse cryo-imaging, a cryoslicing system^45^ equipped with a fluorescence camera (Luca R EMCCD, Andor) was used. For the excitation of HCT116 mice, a 680-nm CW diode laser (B&W Tek, 300 mW) was used; for the 4T1 mouse, a 750-nm CW diode laser (B&W Tek, 300 mW) was used. A 721/42 filter (Chroma) was employed as emission filter for acquiring fluorescence images of the HCT116 mice and an LP 785 filter (Chroma) as emission filter for the 4T1 mouse. For the fluorescence images that were used as ground truth, thin slices of 50 µm from the block tissue were imaged using the cryo-imaging system.

Stitched fluorescence microscopy images were acquired using a Zeiss Axio Imager M2 microscope with a AxioCam MRm monochrome digital camera.

#### Ground truth images

In the case of phantoms, image distortion was considered to be caused mainly by the optical properties of the three areas surrounding the phantom and not by the fluorescent phantom itself. For this reason, the acquired image of the phantom surrounded by air was considered as the ground truth image and it was averaged to suppress the noise. In mouse tissue experiments, we considered the thin slice to be minimally affected by diffusion and therefore to be the closest to the true fluorescence distribution within the specific slice. We chose to acquire slices of 50 µm, so that fluorescence signal would be strong enough.

#### Retrieval of the kernels

Due to the spatial variability of the convolution kernel and the discrete nature of the image, we defined a K(x, y) kernel function, where x, y are the pixel coordinates, to represent the kernels for every pixel within the captured image. To calculate each single kernel, we projected a point-like source onto the sample surface by means of a DLP projector (DLP 3000 Lightcrafter, Texas Instruments, Dallas, USA), and we recorded the reflection image produced. Each reflection image represents the spatially-resolved reflectance of the source and it depends on the optical properties of the sample at this specific point. We considered this image as an approximation for the unknown convolution kernel that essentially blurs the true fluorescence image locally. This point-like source was used to scan the entire area of interest (see Supplementary Video, kernels with green pseudocolor), and all reflection images were recorded for the whole area. As a result, a unique kernel was assigned to each image pixel.

The projector was positioned above the sample at a very small angle (below 5 degrees) such that the projected spots did not appear distorted. Only the red LED channel (624 ± 18 nm) of the projector was utilized since it was the closest to the absorbance and emission peaks of the fluorescence agents in the phantom and mouse tissue. Instead of using the raw reflection image as a kernel, every reflection point image was fitted to a symmetrical, two-dimensional Gaussian curve^48,49^, although more complex models involving sums of Gaussians exist^50^. Each Gaussian curve was considered homogeneous in all directions and the standard deviation value (sigma) was retrieved for every curve.

In order to retrieve values from the whole imaging surface, the latter was scanned by the point-like beam source on a pixel-by-pixel basis^51^. After Gaussian fitting and averaging^51^ of the neighboring pixels, a two-dimensional array of sigma values was formed, containing a sigma value for every pixel of the acquired raw fluorescence image. The reflection images were recorded by means of a second camera, but it is also possible to use the fluorescence imaging camera without any emission filter. Sigma values were adjusted using the same calibration surface for the various experiments, and all the images were co-registered.

To correct for uneven light projection, the reflection image was divided by the reflection image of a homogeneous white surface when all the pixels of the projector were on. To disengage the projected spot from the projection system characteristics, we did the following. We considered that the measured kernel (K_measured_) equals the convolution (Eq. 1) of the raw trace (I_projector_) on the imaging surface with the unknown kernel (K) we were seeking. We assumed that kernel used in the deconvolution scheme (K) depends exclusively on the local optical properties of the tissue.

1 $${{\rm{K}}}_{{\rm{measured}}}\approx {{\rm{I}}}_{{\rm{projector}}}\ast {\rm{K}}$$

In order to estimate the spatially variable I_projector_, we used a homogeneous black semi-reflective surface and repeated the aforementioned scanning procedure to retrieve reflectance images that approximate the I_projector_. Symmetrical Gaussian curves were then fitted in an effective way (coefficient of determination >0.85), and a two-dimensional array with sigma values was generated. Finally, since K_measured_ and I_projector_ were both approximated by Gaussians, we could extract the sigma of the kernel K from the following equation (Eq. 2), which is based on Gaussian convolution properties:

2 $${{\rm{sigma}}}_{{\rm{K}}}=\sqrt{{{\rm{sigma}}}_{{{\rm{K}}}_{{\rm{measured}}}}^{2}-{{\rm{sigma}}}_{{{\rm{I}}}_{{\rm{projector}}}}^{2}}$$

### Data processing

#### Deconvolution

The raw acquired image was considered to be the result of a convolution operation of the true fluorescence image with a kernel that is dependent upon the optical properties of the imaging area. The distortion introduced by the optical imaging system was considered negligible compared to diffusion effects due to light-tissue interactions.

After assigning a sigma value to every pixel, we needed to perform deconvolution for each pixel separately. Nevertheless, a per-pixel deconvolution step would be extremely computationally intensive and, thus, time-consuming. Therefore, we binned the sigma values into *n* equally sized bins and the lowest sigma value of every bin was used to create a new Gaussian kernel which was taken as the input kernel for the upcoming deconvolution step. For the deconvolution step, we elected to perform a Lucy-Richardson iterative restoration method, widely used in other bioimaging applications. We also used a regularization constraint based on total variation to suppress the noise that is amplified during the iterations^33^. Finally, we performed *m* cycles of deconvolution and weighted the results according to Eq. 3:

3 $${{\rm{W}}}_{{\rm{i}}}=1-(\frac{|{\rm{sigma}}(x,y)-{{\rm{sigma}}}_{\mathrm{bin}(i)}|}{\max (\mathrm{sigma})-\,\min (\mathrm{sigma})})$$

where W_i_ is the weight for the *i*th deconvolution of the (x, y) pixel, sigma(x, y) is the corresponding sigma value from the sigma map and sigma_bin(i)_ is the binned sigma value that was used for the construction of the kernel of the *i*th deconvolution.The contribution of each deconvolution result to a given pixel is higher when the sigma value of that pixel is closer to the sigma value of the kernel used to produce the deconvolution result. All computations were performed in MATLAB (Mathworks, USA).

#### Evaluation of performance

We examined the sharpness of the corrected fluorescence images using the Brenner gradient^52^, which is an image differentiation function that can be used as a measure of image focus. The larger the gradient, the sharper the image is. Furthermore, the Root Mean Square (RMS) of the difference between corrected and ground truth images was used as a metric to assess improvement due to the correction process. The lower the value, the more the corrected image resembles the ground truth.

Finally, we compared SAIRC with another deconvolution method that uses regularization and a fixed PSF. This method is based on regularized Wiener deconvolution^53^ and is implemented in the ImageJ plugin FFTJ. For the PSF, we simulated a homogeneous Gaussian kernel based on the average value from the sigma map. The regularization parameter was optimized by visual inspection in order to reach a compromise between blurriness and appearance of artifacts in the final image.

#### Estimation of deconvolution parameters

**Figure 5:**
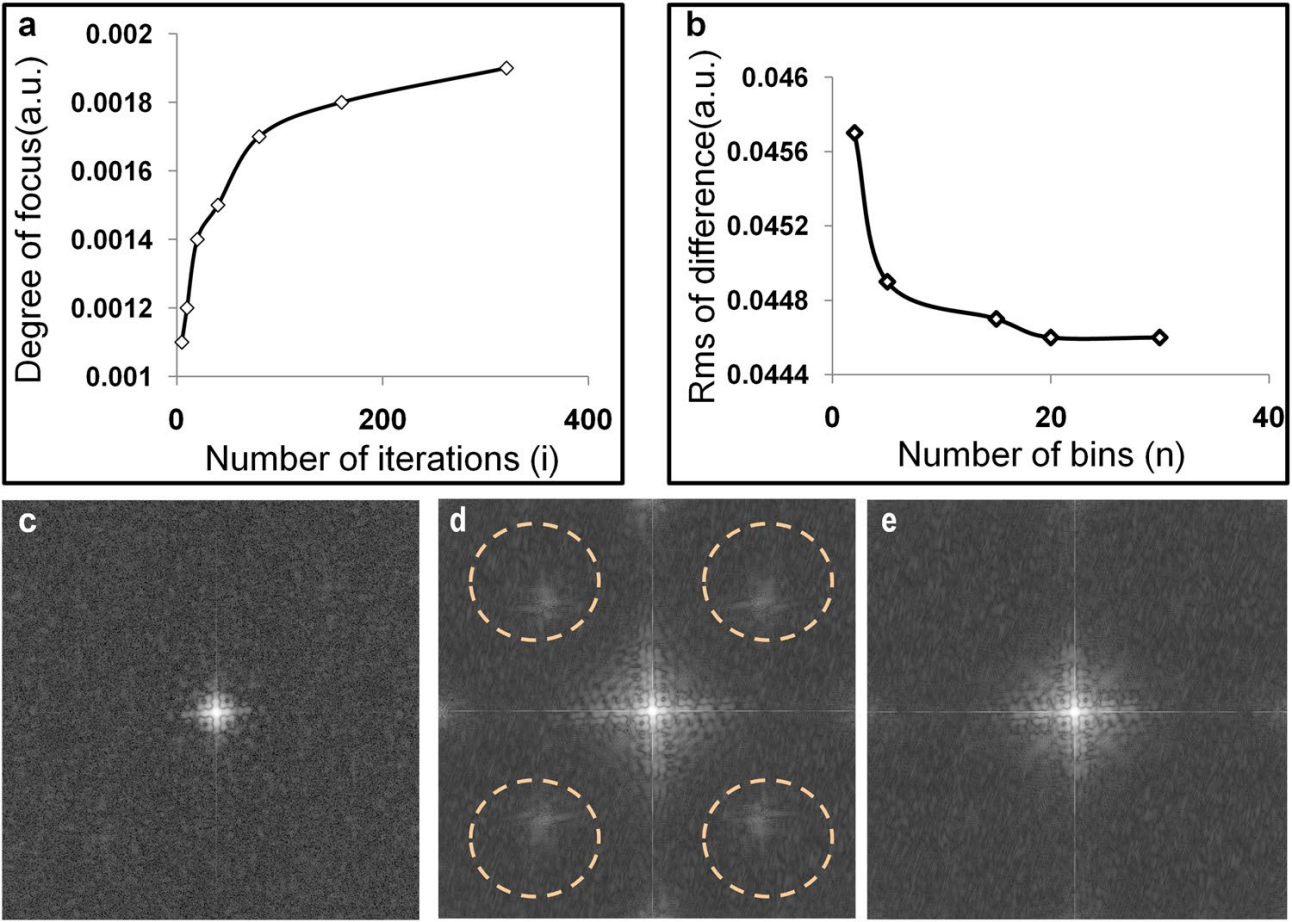
Metrics to estimate optimal parameters for the deconvolution algorithm. (**a**) Evaluation of image sharpness regarding the number of iterations. (**b**) Rms value of the image difference regarding the number of the sigma bins. (**c**) Amplitude Fourier domain of the initial fluorescence image. (**d**) Amplitude Fourier domain of the deconvolved fluorescence image (high λ). (**e**) Amplitude Fourier domain of a deconvolved fluorescence image (suitable λ).

In order to optimize the number of iterations, the number of bins, and the regularization parameter (λ), all of which are inputs in the deconvolution algorithm, we performed tests with different parameter combinations and identified the values that gave the best performance according to quality metrics. We tested the optimal number of iterations by assessing the image sharpness with respect to different iteration numbers. Figure 5a depicts the Brenner’s degree of focus measure regarding the number of iterations with a certain number of bins and λ (*20 bins, λ= 0.02*). We observed a trend of increasing Brenner’s gradient as the iterations increased between 5 and 100. Beyond 100 iterations, the increase in sharpness slowed. Even if the degree of focus was slightly better after 300 iterations, some artifacts started to appear in the image. For this reason, we performed <300 iterations to keep computation time ~1 min without compromising image quality.

To select an appropriate amount of bins for the sigma values, we used as a metric the RMS of the difference between the corrected and ground truth fluorescence images. As observed in Fig. 5b, for a certain number of iterations and λ (iterations = 100, λ = 0.02), the RMS appeared to decrease with increasing number of bins, as expected. After 20 bins, though, the RMS appeared to start saturating, indicating that a large number of bins would not improve the quality of the deconvolution result. Thus, we may minimize the computational time by using only 20 bins.

The regularization parameter λ should be selected in a way that is high enough to suppress the image noise but low enough so it will not impede the restoration process. For high λ, points of very high intensity become more prominent because they are amplified at each iteration^33^. This can be noticed in the Fourier amplitude space of the image. Figure 5c represents the amplitude Fourier space of the raw blurred fluorescence image, while Fig. 5d represents the equivalent for the deconvolved image. We observed that more frequency content was restored after deconvolution, as expected. Nevertheless, we also observed the amplification of other parasitic frequencies (dotted circles in Fig. 5d) as λ increased. For this reason, after inspecting the Fourier space of the deconvolved image, we selected the maximum λ just before the appearance of the parasitic frequencies. For the phantom, we selected λ values around 0.02, whereas for the tissue values around 0.001. The higher value of the phantom parameter can be attributed to the much greater noise in the phantom than in mouse tissue.

## Supplementary information

Supplementary Notes

Supplementary video

## Data Availability

All the data are available by the corresponding author under reasonable request.
