# Supplementary material for: Fluorescence imaging reversion using spatially variant deconvolution
Source: Sci Rep. 2019 Dec 2;9:18123. doi: 10.1038/s41598-019-54578-0 (PMC6889134; doi:10.1038/s41598-019-54578-0)
Supplement: Supplementary file 1 — Supplementary Notes [file 41598_2019_54578_MOESM1_ESM.zip › Supplementary Info/Supplementary Notes.docx]

**Supplementary Notes**

**Fluorescence imaging reversion using spatially variant deconvolution**

Maria Anastasopoulou^1, 2^, Dimitris Gorpas^1, 2^, Maximilian Koch^1, 2^, Evangelos Liapis^1, 2^, Sarah Glasl^2^, Uwe Klemm^2^, Angelos Karlas^1, 2^, Tobias Lasser^3^, Vasilis Ntziachristos^1, 2,*^

^1^ Chair of Biological Imaging and TranslaTUM, Technical University Munich, Munich, 81675, Germany

^2^ Institute of Biological and Medical Imaging, Helmholtz Zentrum München, Neuherberg, 85764, Germany

^3^ Computer Aided Medical Procedures, Technical University Munich, Garching, 85748, Germany

^*^v.ntziachristos@tum.de

1. **SAIRC dependence on wavelength**

Since light scattering is wavelength dependent, one would expect that excitation and emission light influence differently the formation of fluorescence images. Indeed, if the reduced scattering coefficient is approximated as a function of wavelengths, for the most common tissue components, from [1]:

$\mu_{s}^{'}=a\left( \frac{\lambda}{500(nm)} \right)^{-b}$ (4)

this variation can be observed in Suppl. Fig. 1. In equation (4) λ is the wavelength, a is a scaling factor term and b is the ‘scattering power’ term as fully described in [1]. The average values of a and b from [1] for different tissue components (i.e. brain, breast, bone, other soft tissues, other fibrous tissues, fatty tissue) were applied in equation (4). As shown in Suppl. Fig. 1, the difference between 500 nm and 800 nm is more than 40%. However, when excitation and emission wavelengths are relatively close, this difference becomes significantly smaller. For example in the mice bearing HCT116 tumors that express the near-infrared fluorescent protein iRFP720 (Fig. 4), the excitation was centered at 680 nm and emission at 721 nm, while SAIRC was implemented with a Digital Light Processing (DLP) configuration centered at 624 nm. This is translated to a scattering difference of 9% (averaged from all the components) between DLP and excitation and 15% between DLP and emission. Such values are small enough to be neglected during the development of SAIRC and to showcase its potential. Nevertheless, while working towards the optimization and expansion of SAIRC to also incorporate quantitative fluorescence intensity correction, the wavelength-dependent influence of the tissue optical properties to the acquired data will be considered.

**
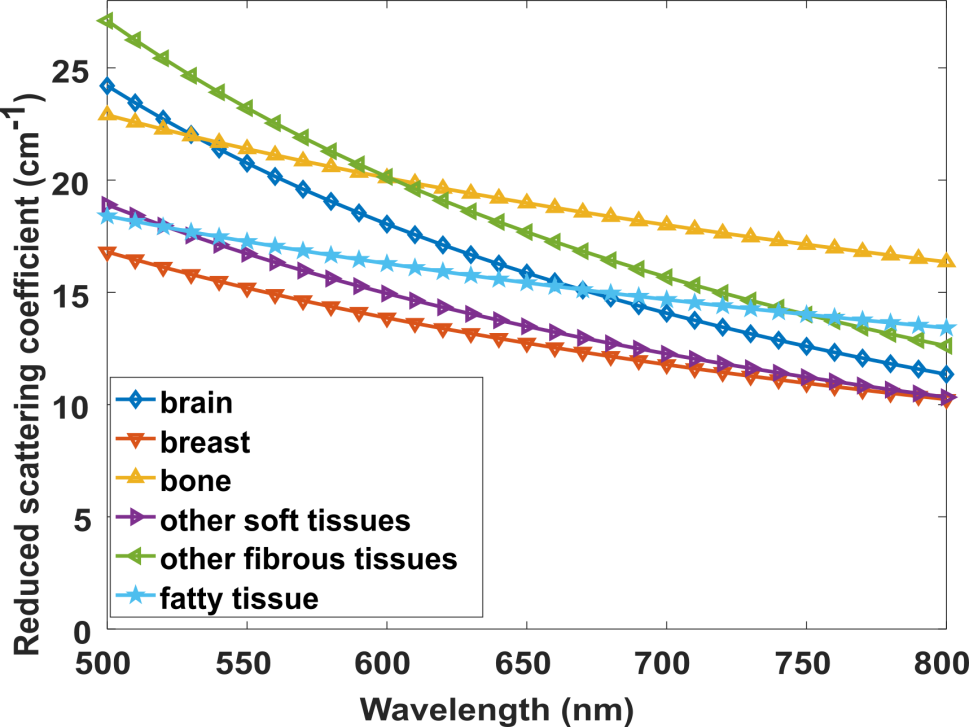
**

Suppl. Fig. 1. Reduced scattering coefficient for different tissues in the near-infrared range.

1. **Faster scanning acquisition**

The long-term objective of this study is the translation of SAIRC in clinical applications. That, however, necessitates significantly faster data acquisition. To this end, imaging and scanning of a grid (Suppl. Fig. 2a) has the potential to significantly reduce acquisition time. This can be further improved by parallelizing the fitting process, in order to simultaneously analyze multiple points, by using a more sensitive camera (i.e. shorter exposure time) and higher computational power. Nevertheless, the preliminary results of Suppl. Fig. 2, that achieved six-fold acquisition time improvement compared to a single point raster scanning in data acquisition are shown.


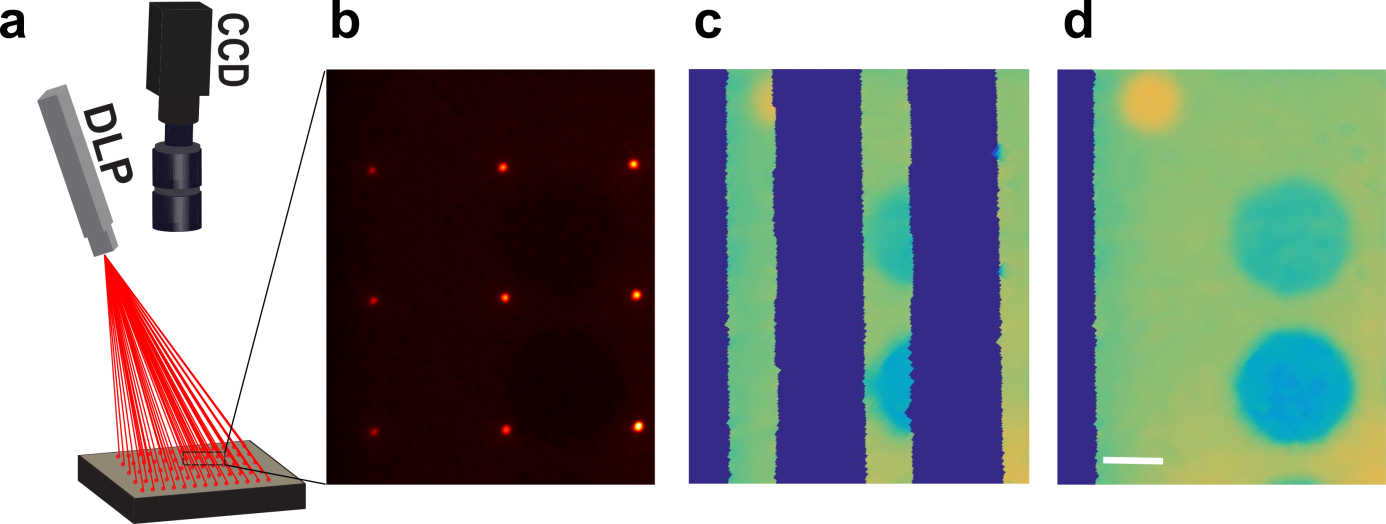


Suppl. Fig. 2. Simultaneous acquisition of multiple points scanned on the sample. A solid phantom with wells of different optical properties [2] is being scanned. (a) Schematic of the DLP projector that projects a grid of points. (b) Projected points on the phantom surface with different optical properties. (c) Fitting and creation of the sigma map for all the grid points simultaneously. (d) Sigma map acquired from the scanning progress. DLP: DLP 3000 Lightcrafter, Texas Instruments, Dallas, USA. CCD: Grasshopper 3, PointGrey, Wilsonville, USA. Scalebar: 5 mm.

1. **Supplementary Video: DLP scanning of a mouse bearing a 4T1 tumor**

This video illustrates the scanning procedure with a single point raster scanning of a cryosectioned mouse bearing a 4T1 tumor, embedded in OCT (Optimal Cutting Temperature) compound, with the DLP projector. Kernel acquisition video is overlaid with green color on a grayscale image of the tissue.

**References**

[1] S. L. Jacques, “Optical Properties of Biological Tissues: A Review,” *Phys. Med. Biol.*, vol. 58, no. 11, pp. R37-61, 2013.

[2] D. Gorpas *et al.*, “Multi-parametric Standardization of Fluorescence Imaging Systems Based on a Composite Phantom,” *IEEE Trans. Biomed. Eng.*, p. 1, 2019.
