# Supplementary figures and images for: Fluorescence imaging reversion using spatially variant deconvolution
Source: Sci Rep. 2019 Dec 2;9:18123. doi: 10.1038/s41598-019-54578-0 (PMC6889134; doi:10.1038/s41598-019-54578-0)

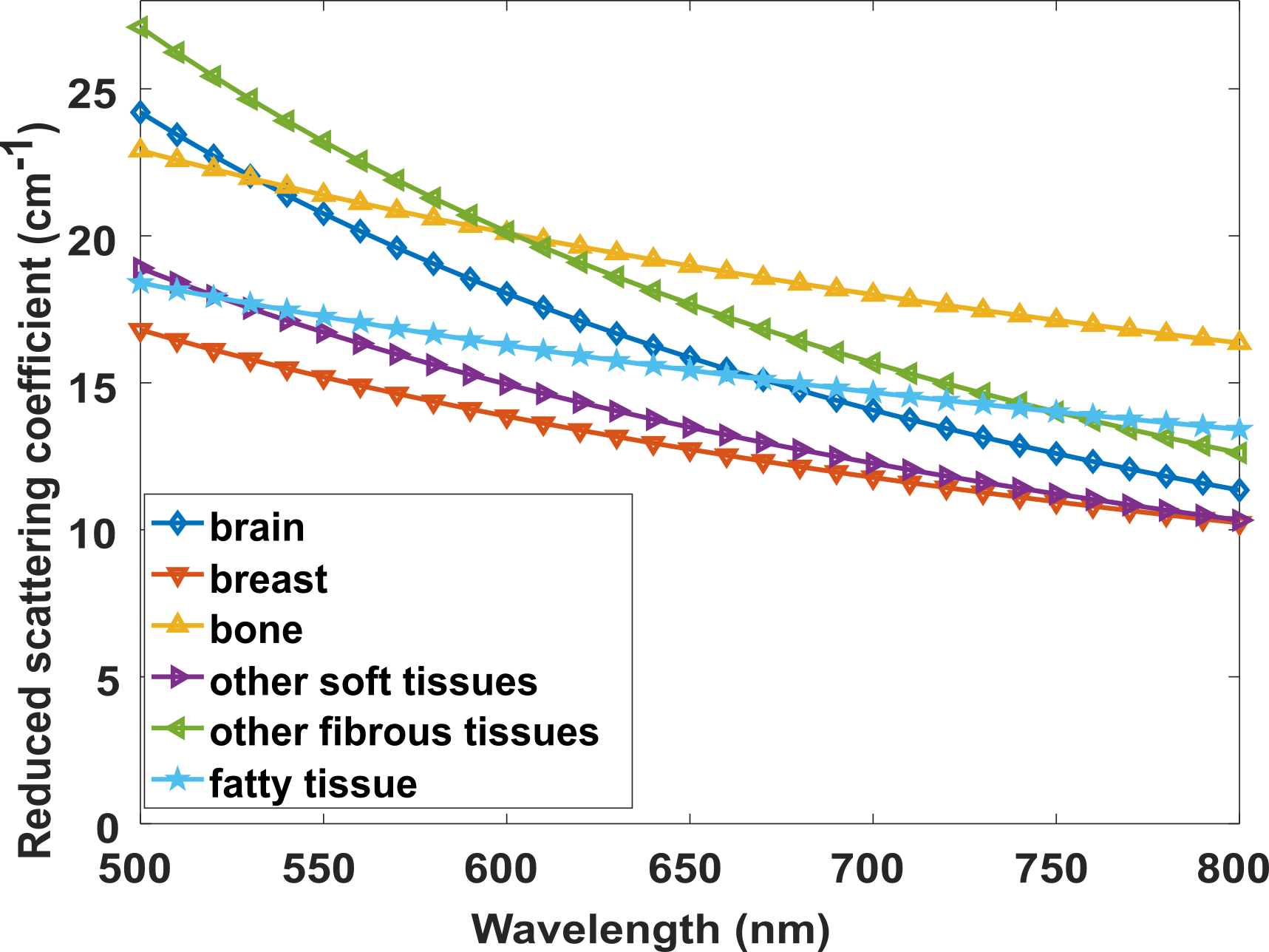

Supplement: Supplementary file 1 — Supplementary Notes [file 41598_2019_54578_MOESM1_ESM.zip › Supplementary Info/Suppl.Fig1.png]

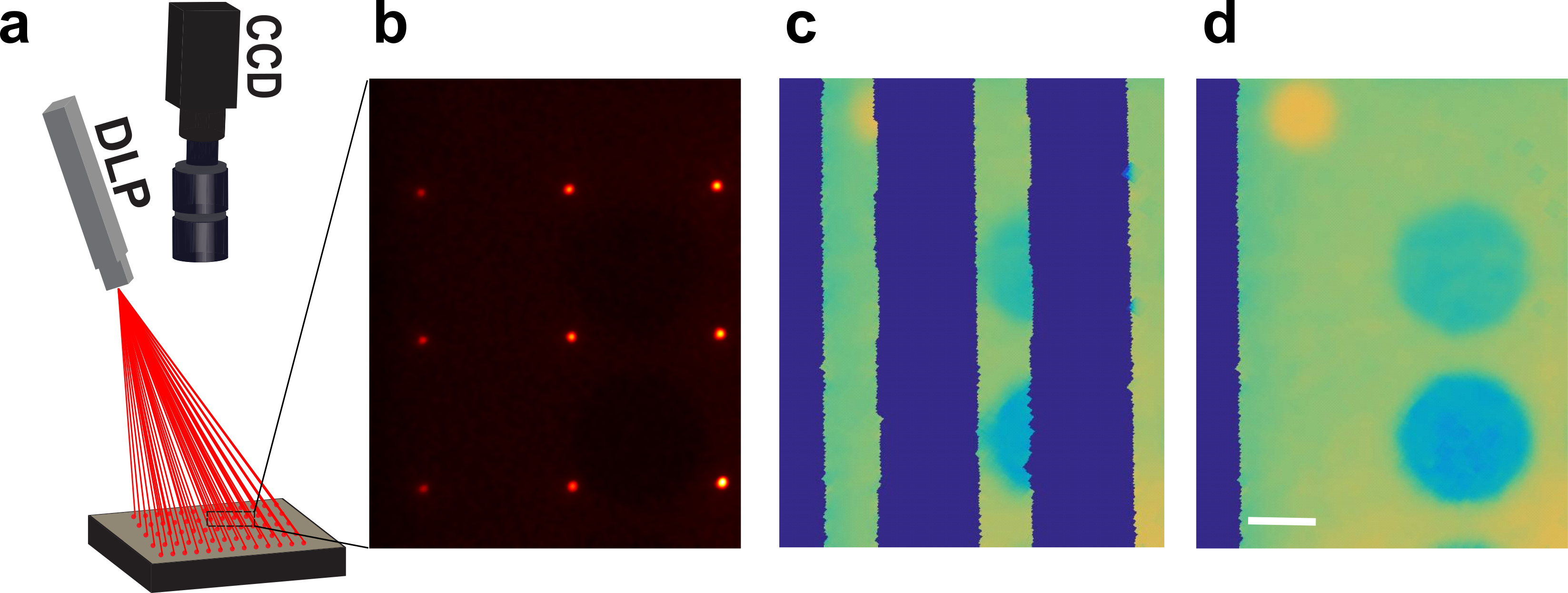

Supplement: Supplementary file 1 — Supplementary Notes [file 41598_2019_54578_MOESM1_ESM.zip › Supplementary Info/Suppl.Fig2.png]
